# Spontaneous Left Main Coronary Artery Dissection in a Male

**DOI:** 10.7759/cureus.60587

**Published:** 2024-05-19

**Authors:** Lakshay Chopra, Joseph Maenza, Chih-Chiun Chang, Syed Muhammad Ibrahim Rashid, Yumiko Kanei

**Affiliations:** 1 Internal Medicine, Icahn School of Medicine at Mount Sinai, New York, USA; 2 Cardiology, Icahn School of Medicine at Mount Sinai, New York, USA

**Keywords:** primary percutaneous coronary intervention (pci), male patient, atypical spontaneous coronary artery dissection, scad management, left main coronary artery disease (lmcad)

## Abstract

Spontaneous coronary artery dissection (SCAD) is one of the causes of acute coronary syndrome (ACS) that is increasingly recognized in young to middle-aged women without typical coronary risk factors. This case report describes a 46-year-old male with a rare presentation of SCAD involving the left main (LM) coronary artery. The patient underwent an emergency coronary angiogram for high-risk ACS and had percutaneous coronary intervention (PCI) of LM due to active ischemia and hemodynamic instability. The extension of intramural hematoma after the LM coronary artery stent confirmed the initial suspicion of SCAD. The diagnosis of SCAD is crucial, as its management differs from other causes of ACS. Coronary angiography is the gold standard for diagnosing SCAD, with adjunctive imaging using optical coherence tomography (OCT) and intravascular ultrasound (IVUS). In this patient, his physical examination findings and further imaging raised a suspicion for systemic connective tissue disease. Genetic analysis was executed, but no reportable variants in any of the 29 genes studied were identified. This case highlights the importance of recognizing SCAD as a potential cause of ACS even in men and emphasizes the findings during coronary angiography that can aid in an accurate diagnosis and appropriate management.

## Introduction

Spontaneous coronary artery dissection (SCAD) is mediated by an intimal tear or bleeding in the media of the coronary artery, creating a false lumen and subsequent intramural hematoma. This hematoma bulges into the lumen and causes coronary vessel obstruction [1,2]. SCAD has been increasingly recognized as a cause of acute myocardial infarction (AMI), with recent studies suggesting that SCAD accounts for 0.7-4% of AMI [3-7]. SCAD has a female preponderance, with 90% of the cases seen in women [8]. Arteriopathies like fibromuscular dysplasia are associated with SCAD. Hormonal changes during pregnancy and the early postpartum period have also been suggested as possible factors contributing to the higher incidence of SCAD in women [2,8-10].

SCAD typically occurs in younger patients in whom classical atherosclerotic risk factors are often absent. The adjusted mortality in patients with SCAD is equivalent to other causes of AMI [6]. We present a SCAD of the left main (LM) artery in a male patient and discuss the critical differences in the management approach to AMI caused by SCAD, aiming to increase the awareness of SCAD in men.

## Case presentation

**Figure 1:**
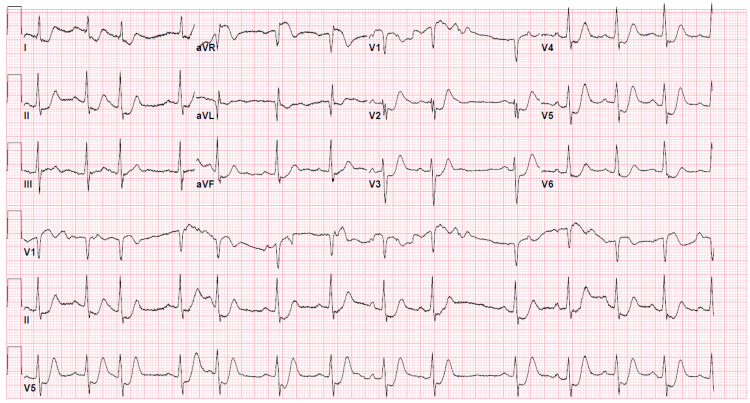
EKG showing ST elevation in lead aVR and diffuse ST depressions in the anterolateral and inferior leads (I, II, aVF, and V2-V6)

A 46-year-old male was brought in by an ambulance with severe left substernal chest pain associated with diaphoresis and dyspnea, which started 30 minutes before the presentation while ambulating. The electrocardiogram (EKG) showed diffuse ST depressions in leads I, II, aVF, V2-V6, and ST elevation in aVR (Figure 1). On presentation, he was in respiratory distress, diaphoretic, and reporting active chest pain. His blood pressure was 89/54 mmHg, heart rate 93 bpm, respiratory rate 26/min, and O_2_ saturation of 89% on room air. He was given aspirin 325 mg, ticagrelor 180 mg, heparin bolus placed, on bilevel-positive airway pressure support, started on a dopamine drip, and underwent an emergency coronary angiogram.

The patient had a previous medical history of hypertension, obstructive sleep apnea, and atraumatic retinal detachment at age 42. In addition, there was a family history of myocardial infarction in his maternal uncle at age 40 and in two paternal uncles at unspecified ages. His home medications included amlodipine and hydrochlorothiazide. He denied any history of drug use.

**Figure 2:**
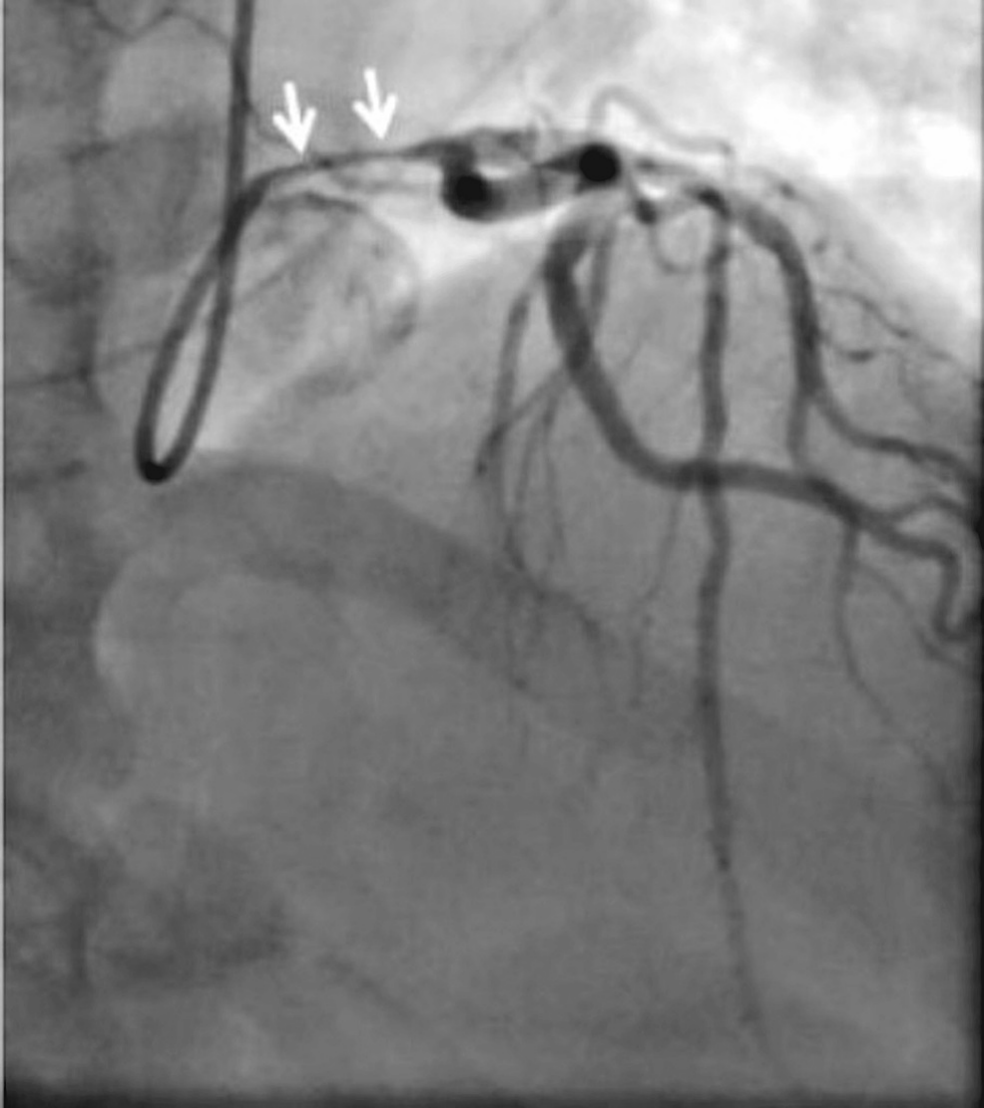
Dissection of the left main coronary artery with intramural hematoma, causing 90% occlusion from the osmium to distal segments.

**Figure 3:**
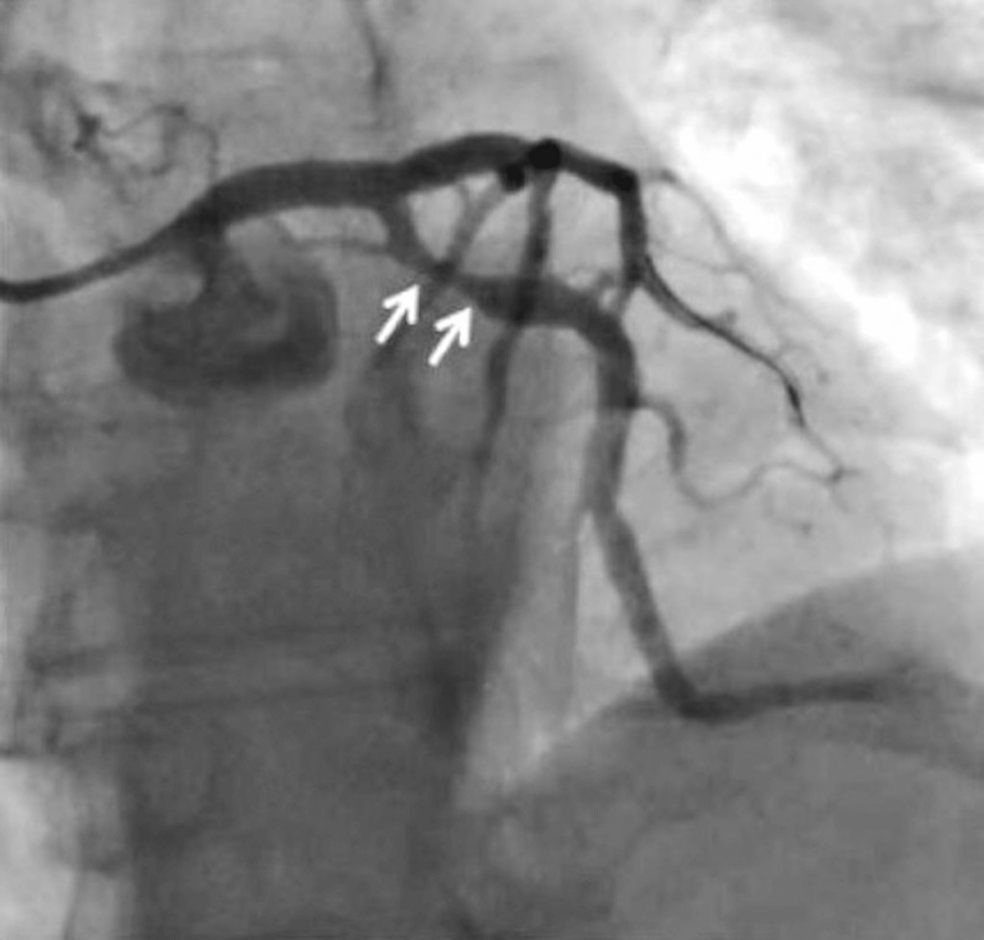
The hematoma appears to have extended into the LCx artery after stent placement in the left main coronary artery.

**Figure 4:**
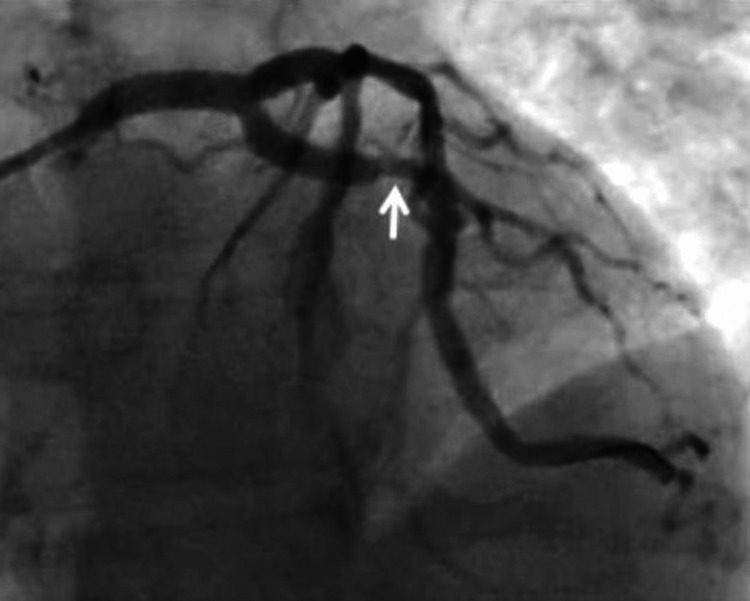
The hematoma appeared to have extended further distally after stents were placed in the LCx.

Coronary angiography showed 90% stenosis of the left main (LM) coronary artery from the ostium to the distal (Figure 2). With his cardiogenic shock, immediate percutaneous coronary intervention (PCI) was performed with a 4.0 mm x 20 mm everolimus-eluting stent (Promus®). The pre-lesion TIMI flow was 1 and post TIMI flow was documented as 3. With intravascular ultrasound (IVUS) guidance, the stent was post-dilated with a 5.0 mm non-compliant balloon to 14 atmospheres. After post-dilatation, a new lesion was noted in the proximal left circumflex artery (LCx), which was thought to be the extension of intramural hematoma, and two additional everolimus-eluting stents were placed, one in the proximal LCx and one in LCx branch OM1 (Figures 3, 4). An intra-aortic balloon pump (IABP) was placed for hemodynamic support. The first troponin I collected at the time of presentation was normal (0.022 ng/ml; reference range <0.031 ng/ml), which rose to the peak of 250 ng/ml. The patient was admitted to the cardiac intensive care unit, he was weaned off the dopamine drip over the next two days, and IABP was removed. He was medically optimized and discharged on day 7 in stable condition. A transthoracic echocardiography (TTE) before discharge showed a normal left ventricular size and ejection fraction without regional wall motion abnormalities.

On physical examination, he was noted to have joint hyper-mobility hypertelorism, broad-based uvula with a possible cleft, sandal toe finding with arachnodactyly of toes, and skin hyperextensibility. Given the prior history of atraumatic retinal detachment and signs suggestive of underlying connective tissue disorder, he was recommended to follow up with a cardiac genetic specialist. His discharge medications included aspirin, ticagrelor, atorvastatin, metoprolol, and lisinopril.

**Figure 5:**
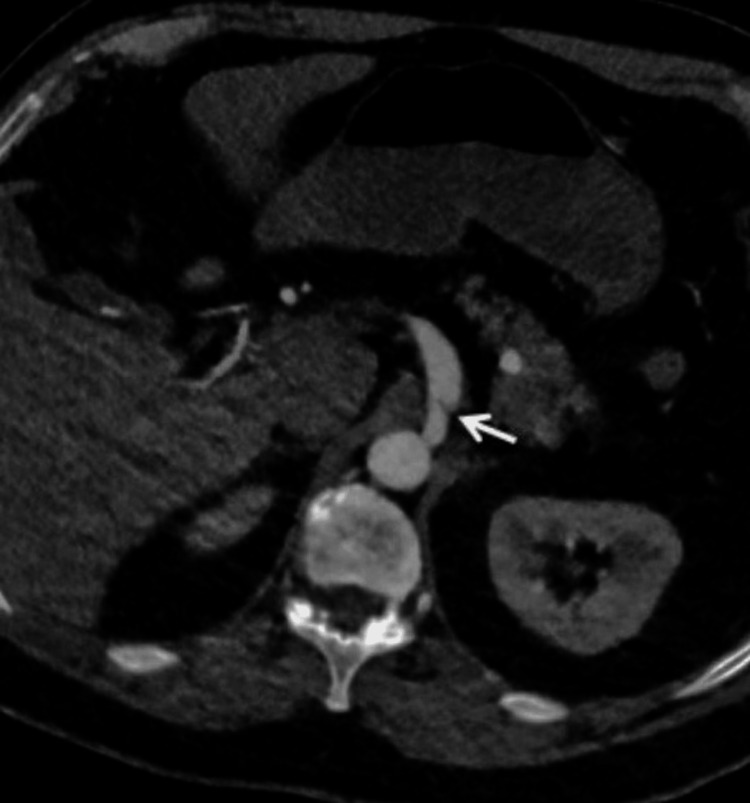
Aneurysmal dilatation of the celiac artery with a proximal focal dissection.

About a month after discharge, he developed epigastric discomfort radiating to the left chest. He underwent cardiac catheterization, which showed patent LM and LCx stents. A computed tomography (CT) of the chest with intravenous contrast, performed to rule out pulmonary embolism, showed an incidental finding of aneurysmal dilatation of the celiac artery with a proximal focal dissection (Figure 5). The patient underwent additional imaging.

**Figure 6:**
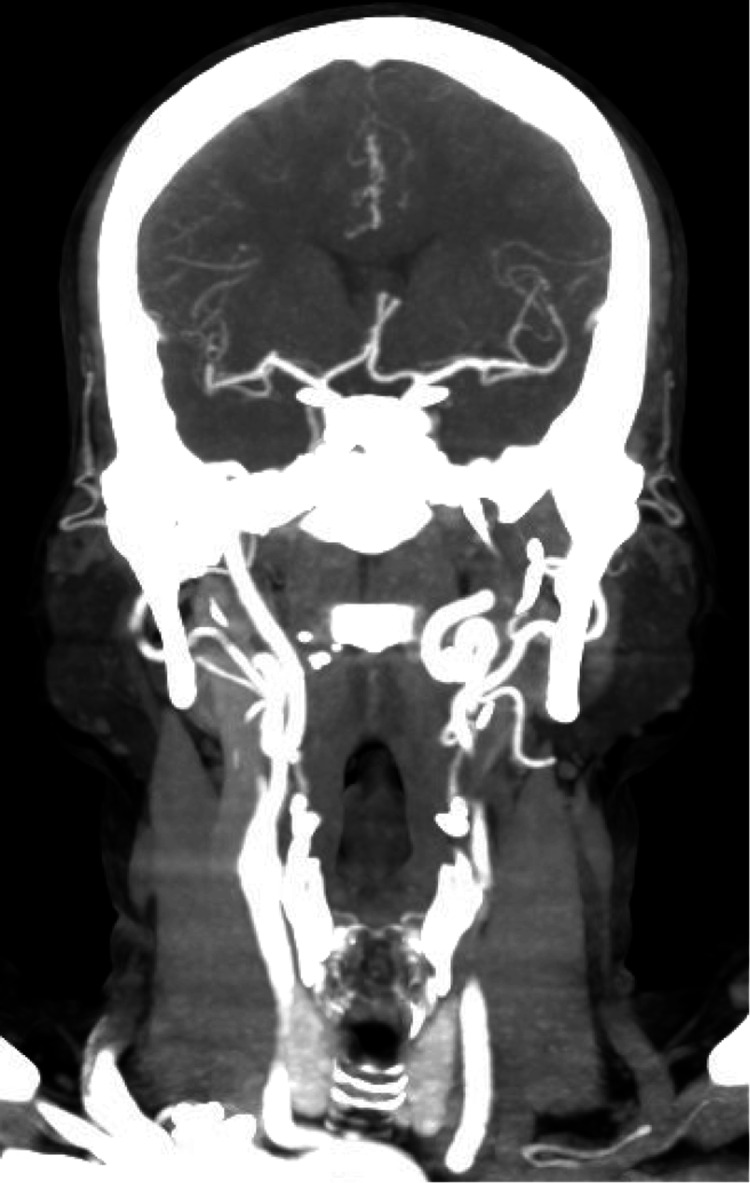
CT angiography showing marked elongation and tortuosity of the bilateral internal carotid arteries

**Figure 7:**
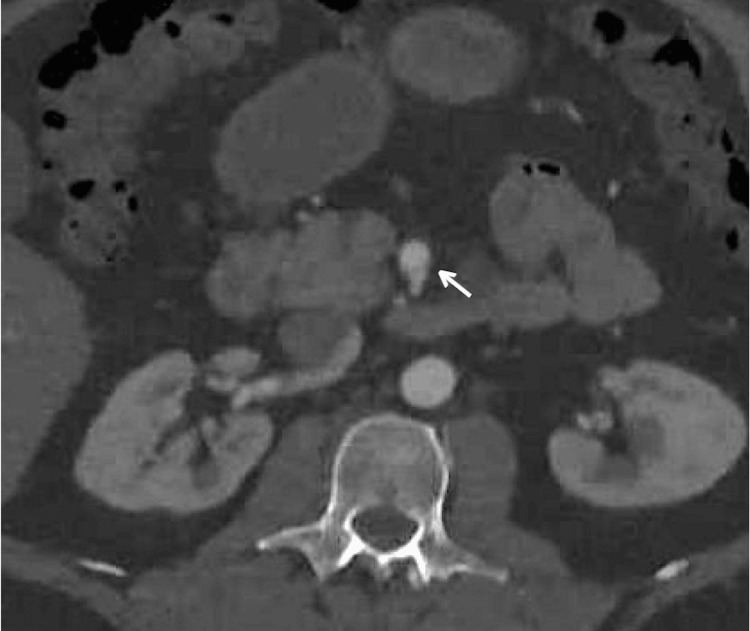
Dissection of the proximal to the mid-superior mesenteric artery

The head and neck CT angiography showed marked elongation and tortuosity of the bilateral internal carotid arteries (Figure 6). CT of the chest, abdomen, and pelvis was significant for diffuse fusiform aneurysm, dissection of the proximal to the mid-superior mesenteric artery, and the previously seen celiac artery dissection (Figure 7).

The patient's physical examination and imaging did not meet the clinical criteria for Marfan, Loeys-Dietz, or vascular Ehlers-Danlos syndromes; nevertheless, these findings raised suspicion for a potential underlying inherited arteriopathy. Options for genetic analysis were considered, and the Invitae© aortopathy comprehensive panel was sent after discussion with the patient [11].

## Discussion

This is a unique case of SCAD in a male patient with LM coronary artery involvement, causing acute coronary syndrome and cardiogenic shock requiring emergent PCI. The diagnosis of SCAD is made by pattern recognition during coronary angiography with optical coherence tomography (OCT) and intravascular ultrasound (IVUS) used for confirmation in certain cases. The accurate diagnosis of SCAD is critical since its management differs from other causes of acute coronary syndrome.

The optimal treatment strategy for SCAD remains controversial. In most patients with coronary artery lesions due to SCAD, they heal spontaneously; thus, a conservative approach is preferred in hemodynamically stable patients [2,12]. PCI in SCAD is challenging and can have a higher incidence of unsuccessful PCI. It is associated with a higher risk of complications, such as hematoma propagation, dissection extension, and false lumen wiring. However, PCI is required in unstable patients with LM coronary artery involvement and those with ongoing ischemia or hemodynamic compromise [2,13]. In this case, PCI was immediately performed as a life-saving treatment in an unstable patient with severe LM artery obstruction. Since he was a male patient with some atherosclerotic risk factors, SCAD was not a leading diagnosis prior to PCI and IVUS.

The recurrence of SCAD is not rare. In the Vancouver SCAD cohort, the recurrence rate was 10.4% over a median follow-up of 3.1 years. Hypertension was associated with an increased incidence of SCAD, and beta-blocker use was associated with an approximately 70% reduction in the risk of recurrent SCAD [14]. Aspirin and beta-blockers are typically used for long-term therapy after SCAD. The second anti-platelet agent is added if the lesion undergoes PCI revascularization, but the benefit of a second anti-platelet agent in the absence of intervention is not clear. Systemic anticoagulation carries a theoretical concern for intramural hematoma extension, and it should be discontinued once the diagnosis of SCAD is made [2].

Twenty-nine genes were analyzed; the list of genes and the clinical syndrome they are involved in is given in Table 1. No reportable variants were identified in any of the genes analyzed.

**Table 1:** Common genes associated with spontaneous coronary artery dissection (SCAD)

Clinical disorder	Genes involved	Description
Arterial tortuosity syndrome	SLC2A10	This syndrome is characterized by widespread tortuosity and stenosis of the aorta and mid-sized arteries. Physical examination findings include hyperextensible skin, joint hypermobility, and characteristic skeletal findings [15].
Congenital contractural arachnodactyly	FBN2	Arachnodactyly, flexion contractures, and marfanoid habitus characterize this syndrome. Cardiovascular and/or gastrointestinal anomalies are seen on the severe end of the spectrum of Congenital contractural arachnodactyly [16].
Ehlers-Danlos syndrome	COL5A1, COL5A2, COL3A1, PLOD1, BGN	They are a heterogeneous group of connective tissue disorders, commonly presenting with joint hypermobility, hyperextensible skin, abnormal wound healing, and easy bruising. Varying degrees of manifestations are found throughout the body in different types of Ehlers-Danlos syndrome [17].
Loeys-Dietz syndrome	TGFBR2, TGFBR1, SMAD3, TGFB2, TGFB3, SMAD2, SMAD4 SKI	Abnormal tortuosity, aneurysms, and dissections of the blood vessels characterize this syndrome. Joint hypermobility, skin hyperextensibility, craniosynostosis, arachnodactyly, and scoliosis can also be seen [18].
Marfan syndrome	FBN1	Primarily characterized by skeletal, ocular, and cardiovascular abnormalities. Individuals typically exhibit tall stature, long limbs, and arachnodactyly. Cardiovascular complications may include aortic dilation, dissection, and mitral valve prolapse. Ocular issues often involve lens dislocation. While the phenotype varies, these features are indicative of the syndrome’s impact on connective tissue throughout the body [19].
Thoracic aortic aneurysms and dissections	ACTA2, MYH11, MYLK, PRKG1, FOXE3, LOX	Characterized by abnormalities in the thoracic aorta. Familial thoracic aortic aneurysms and dissections can lead to life-threatening events. Surveillance and prophylactic intervention are recommended [20].
Weill-Marchesani syndrome, stiff skin syndrome	ADAMTS10	Conditions causing short stature, brachydactyly, ocular anomalies, and hard, thick skin developing in infancy [21].
Homocystinuria	CBS	An inherited disorder with high homocysteine levels, causing developmental delays and a tendency to form blood clots [22].
Otopalatodigital spectrum disorders	FLNA	Disorders with a variety of phenotypes, including skeletal, cardiac, and neurologic symptoms [23].
X-linked intellectual disability	MED12	Mutations in this gene have been implicated in syndromes presenting with intellectual disability, developmental delays, and distinctive facial features [24].
Fibulinopathies	EFEMP2	Associated with disorders such as cutis laxa which affect the skin, lungs, and vascular system, leading to inelastic and sagging skin [25].
Systemic sclerosis	NOTCH1, MFAP5	These genes are linked to systemic sclerosis and aneurysms, with NOTCH1 also being implicated in aortic valve disease [26].

According to a prospective study involving 750 patients, fibromuscular dysplasia is strongly associated with SCAD. The peripartum period, systemic inflammatory diseases, and concomitant connective tissue disorders have also been identified as risk factors for SCAD.

Notably, in two-thirds of SCAD patients, a physical or emotional trigger can be identified, with physical triggers being more common in men [8,27]. In this case, no identifiable trigger was found, but the patient had extra-coronary manifestations and a high suspicion of a systemic disease process. The current understanding regarding the utility of genetic testing, or even the identification of variants, in SCAD, is rather limited. With the growing knowledge of SCAD, there is hope for the development of actionable genetic risk scores, which could serve as crucial tools in detecting potentially harmful disease manifestations in other organ systems and facilitate the screening of at-risk relatives. Our recommendations for the patient incorporated the current consensus of genetic analysis in situations where there is a clinical suspicion of an underlying inherited arteriopathy [2].

Despite the extensive genetic analysis, the negative findings suggest that other genetic or environmental factors might contribute to the pathogenesis of SCAD. This observation underscores the complexity and heterogeneity of the disease and indicates that SCAD may be the result of multifactorial influences that extend beyond the currently known genetic markers.

Given the absence of identifiable mutations in the known genes, this case suggests the possibility of as-yet unidentified genetic factors contributing to the pathogenesis of SCAD. The need for a more comprehensive genetic analysis is evident, and there is a clear imperative to develop better risk stratification models to predict and manage SCAD effectively. This case emphasizes the importance of ongoing surveillance and research to identify new genetic markers and mechanisms underlying SCAD. The patient's counseling for regular follow-up and the advice provided to first-degree relatives to seek evaluation reflect a personalized approach to managing conditions with unclear genetic contributions. Future research should focus on expanding the genetic panels used for SCAD and integrating multi-omic approaches to uncover the full spectrum of genetic and environmental factors contributing to its occurrence.

## Conclusions

This case highlights the importance of recognizing SCAD as a potential cause of acute coronary syndrome in men and emphasizes the need to differentiate it from other causes of AMI. PCI is associated with a higher risk of dissection extension and false lumen stenting. Consequently, conservative management is preferred in SCAD, and PCI is reserved as an immediate life-saving treatment in unstable cases. The presence of extra-coronary manifestations in our patient indicated the potential existence of an underlying systemic connective tissue disease, but the genetic analysis did not add any diagnosis. This inconclusive result of genetic testing might reflect the current limitations in our understanding of SCAD. This helps to underscore the need for enhanced genetic models and more extensive research to identify additional genetic markers that may contribute to SCAD. As the recognition of this condition continues to increase, our understanding and management strategies will evolve. This case not only contributes to the growing body of SCAD literature but also calls for a concerted effort to further elucidate the genetic landscape of this complex condition. The ongoing research and development of better risk stratification models will be crucial in improving the diagnosis, treatment, and prognosis of patients with SCAD.
